# Efficacy and safety of parathyroid hormone analogs therapy on hypoparathyroidism: a meta-analysis

**DOI:** 10.3389/fendo.2026.1847971

**Published:** 2026-07-02

**Authors:** Siting Li, Yuanfang Zhang, Li Zhao, Chao Ma

**Affiliations:** 1 Department of Nuclear Medicine, Shanghai Tenth People’s Hospital, School of Medicine, Tongji University, Shanghai, China; 2 Department of Neurology, Shanghai Tenth People’s Hospital, School of Medicine, Tongji University, Shanghai, China; 3 Department of Nuclear Medicine, Qingdao Central Hospital, University of Health and Rehabilitation Sciences, Qingdao, China

**Keywords:** health-related quality of life, hypoparathyroidism, parathyroid hormone analogs, serum calcium, serum phosphorus

## Abstract

**Purpose:**

To assess the efficacy and safety of parathyroid hormone(PTH) analogs alone as compared with the conventional therapy on HypoPTH, and assess its emphasis on patients’ health-related quality of life (HRQoL).

**Methods:**

Database (PubMed, Web of Science, Embase and Cochrane Library) were systematically searched until February 30, 2026. The primary outcomes were serum calcium and serum phosphate, while the secondary outcomes included 24-hour urinary calcium excretion, serum 25(OH)D, serum 1,25-dihydroxyvitamin D, calcium phosphate product, estimated glomerular filtration rate (eGFR), adverse events, and HRQoL. Meta-analysis was conducted using RevMan 5.4 and STATA 17.0.

**Results:**

Eleven studies were included. Compared to conventional therapy, PTH analogs therapy showed no difference in serum calcium (MD = -0.02 mmol/L; 95% CI, -0.14 to 0.11 mmol/L), serum phosphorus (MD = 0.08 mmol/L; 95% CI, -0.05 to 0.20 mmol/L) and 24-hour urinary calcium excretion (MD = 1.00 mmol; 95% CI, -1.84 to 3.84 mmol). PTH analogs decreased 25(OH) vitamin D, increased 1,25(OH)_2_ vitamin D and eGFR. Additionally, PTH analogs therapy significantly improved HRQoL as measured by the Short Form 36 (SF-36) Health Survey Questionnaire (MD = -7.35; 95% CI, -8.37 to -6.33).

**Conclusion:**

In addition to the comparable control of serum calcium and serum phosphorus levels to conventional therapy, limited data indicate that PTH analogs treatment may be better in regulating the serum vitamin D and maintaining the eGFR for patients with HypoPTH. PTH analogs therapy also improves patients’ HRQoL.

**Systematic Review Registration:**

https://www.crd.york.ac.uk/PROSPERO/, identifier CRD420251089112.

## Introduction

Hypoparathyroidism (HypoPTH) is a disorder of calcium and phosphorus metabolism caused by reduced secretion of parathyroid hormone (PTH) or obstruction of its action. The main causes include iatrogenic injury (thyroid/parathyroid surgery accounts for over 75% of adult cases), autoimmune diseases (such as autoimmune polyendocrine syndrome), genetic defects (such as 22q11.2 deletion syndrome), and abnormal magnesium metabolism (1). As a complex endocrine syndrome, HypoPTH not only leads to electrolyte imbalances such as hypocalcemia and hyperphosphatemia but also adversely affects bone health, nervous system function, muscle activity, and the homeostasis of the cardiovascular system. Patients are prone to a series of complications, including neuromuscular abnormalities, abnormal bone turnover, ischemic heart disease, arrhythmias, and cataracts (2, 3).

Currently, conventional therapy mainly focuses on calcium supplementation and active vitamin D (calcitriol), and thiazide diuretics are used as an adjuvant when necessary to reduce urinary calcium excretion. Most patients can maintain normal or near-normal levels of calcium and phosphorus metabolism through this treatment, without obvious clinical symptoms. However, postoperative patients often require high doses of calcium and active vitamin D supplements, and the required doses tend to increase progressively with the progression of the disease. Under long-term treatment, the demand for medications fluctuates. Many patients have poor control of hypocalcemia, the phosphorus concentration becomes abnormal, and the burden on renal filtration is also increased. Additionally, concerns have been raised about complications such as hypercalciuria, basal ganglia calcification, nephrolithiasis, renal insufficiency, and nephrocalcinosis (2, 4).

As an exogenous PTH preparation, PTH analogs has been gradually applied in the treatment of HypoPTH in recent years (5–7). At present, there are three drugs used in PTH therapy: recombinant human parathyroid hormone (1-34) (rhPTH(1-34), teriparatide), rhPTH(1-84) (Natpara) and TransCon PTH (palopegteriparatide, YORVIPATH). RhPTH (1-34), a truncated recombinant hormone, has the same molecular structure as the active fragment (1-34) of endogenous PTH in the human body (8–10). RhPTH(1-84) is a full-length recombinant hormone with a longer half-life. It only needs to be administered once a day to improve hypocalcemia. It was approved by the U.S. Food and Drug Administration (FDA) in 2015 for the long-term management of chronic HypoPTH in adults and by the European Medicines Agency (EMA) in 2017, and has later been launched in some European countries. TransCon PTH is a long-acting PTH replacement therapy that utilizes TransCon technology to conjugate an inactive PTH(1-34) prodrug with a polyethylene glycol (PEG) carrier. Following once-daily administration, active PTH(1-34) is slowly released *in vivo*, achieving sTable 24-hour plasma concentrations. Currently, more and more clinical studies have applied PTH analogs in patients with HypoPTH, and achieved good efficacy and safety combined with the supplements. However, there is a paucity of research comparing the therapeutic effects of PTH analogs alone against the conventional therapy on HypoPTH, as well as its impact on patients’ health-related quality of life (HRQoL).

## Materials and methods

### Data sources and searches

This study was conducted in accordance with the Preferred Reporting Items for Systematic Reviews and Meta-Analyses (PRISMA) statement. The literature data were retrieved from the PubMed, Web of Science, Embase and Cochrane Library databases between January and February 2026. The following search terms were used: ((hypoparathyroidism) OR (HypoPTH) OR (HypoPT)) AND ((Parathyroid Hormone analogs) OR (recombinant human Parathyroid Hormone) OR (PTH analogs) OR (rhPTH) OR (rhPTH (1-34)) OR (rhPTH (1-84)) OR (TransCon PTH) OR (PTH) OR (PTH (1-34)) OR (PTH (1-84)) OR (Teriparatide) OR (Natpara) OR (YORVIPATH)). A total of 4,988 articles published between 1950 and 2026 (spanning 76 years) were identified through this search strategy.

### Eligibility criteria

1. Study types: Open-label prospective trials, retrospective trials and randomized controlled trials (RCTs);
2. Publication time: January 1, 1996, to February 28, 2026;
3. Study content: PTH analogs therapy (rhPTH (1-34), rhPTH (1-84) or TransCon PTH) for children and adults with chronic HypoPTH;
4. Academic impact and authority: The included literature must demonstrate a certain academic influence and authority. Influence is measured by the citation count over the past five years (exceeding the field median of the same period). Meanwhile, only articles published in Journal Citation Reports (JCR) Q1, Q2 or Q3 journals are eligible; Studies with excessive self-citations and non-peer-reviewed publications are excluded;
5. Quantitative outcomes: Reports must include at least one quantitative outcome that this study focuses on.

### Exclusion criteria

1. Letters, reviews, case reports, conference abstracts, commentaries, and expert opinions;
2. Duplicated studies: Studies with duplicate publications (including overlapping datasets or redundant analyses);
3. Methodological limitations: Studies with significant design flaws (inadequate controls, selection bias) or incomplete data reporting.

### Literature screening and data extraction

Literature screening and data extraction were independently completed by two researchers and cross-checked with each other. In case of disagreements, the two researchers would first negotiate together. If consensus could not be reached, a third reviewer would be consulted to resolve any questions regarding the eligibility of the studies. The following information was extracted from eligible studies: the first author’s name, publication year, research center, type of study design, number of participants in each group, duration of the study, intervention measures, and study outcomes (the effects of the treatment with PTH analogs on the serum calcium concentration, serum phosphorus concentration, 24-hour urinary calcium excretion, active vitamin D concentration, calcium-phosphorus product, estimated glomerular filtration rate (eGFR), adverse events, and HRQoL in patients with HypoPTH).

For overlapping populations, data were extracted only from the most recent and complete studies to avoid duplication of patients. For extended studies or those that included patients previously treated with PTH, data were extracted only when new baseline parameters were provided. Baseline and post-treatment data values were extracted according to the reports. For continuous outcomes, the mean (MEAN) and standard deviation (SD) are available. When studies reported only standard errors, interquartile ranges (Q1, Q3), or raw data, the mean and standard deviation were calculated using the estmeansd package in R. Subsequently, study quality was assessed using the Cochrane Risk of Bias 2.0 (RoB 2.0) tool for RCTs and the Risk of Bias in Non-Randomized Studies of Interventions (ROBINS-I) tool for non-RCTs to determine data reliability, high-quality studies were retained while low-quality studies were excluded. For studies with substantial missing data, exclusion decisions were made in conjunction with their quality assessment results.

### Data synthesis and statistical analysis

The primary outcomes of this study were serum calcium levels and serum phosphate levels, while the secondary outcomes included 24-hour urinary calcium excretion, serum 25(OH)D concentration, serum 1,25-dihydroxyvitamin D concentration, calcium phosphate product, eGFR, adverse events, and HRQoL. HRQoL was evaluated by the scores of the Short Form 36 Health Survey Questionnaire (SF-36 scale). The questionnaire consists of 36 items and is divided into 8 dimensions: Physical Functioning (PF), Role Physical (RP), Bodily Pain (BP), General Health (GH), Vitality (VT), Social Functioning (SF), Role Emotional (RE), and Mental Health (MH). The higher score means the better HRQoL.

This study employed meta-analysis methods to comprehensively assess data heterogeneity and effect sizes. Data were analyzed with the statistical software packages Review Manager 5.4 and STATA 17.0. Heterogeneity was evaluated using Cochran’s Q statistic (a weighted sum of variances in study estimates) and the I² index (the percentage of variation between studies). A Q statistic p-value < 0.1 or I² > 50% indicated the presence of heterogeneity, and the analysis model was selected accordingly (the DerSimonian-Laird random-effects model was used in the presence of heterogeneity, while the Mantel-Haenszel fixed-effects model was applied otherwise). To pool continuous data (mean and standard deviation), the mean difference (MD) between post-values and pre-values and its 95% confidence interval (CI) were calculated according to a random-effects model. The standardized mean difference (SMD) was used as the effect size indicator representing the efficacy of PTH analogs therapy for HypoPTH. All analyses employed two-tailed tests, with a p-value <0.05 considered statistically significant, and results were presented in forest plots. In addition, the funnel plot was used to test the publication bias of this study, and the leave-one-out method was used for the sensitivity analysis. When there were enough studies, Egger’s test and Begg’s test were also performed.

## Results

### Study selection

**Figure 1:**
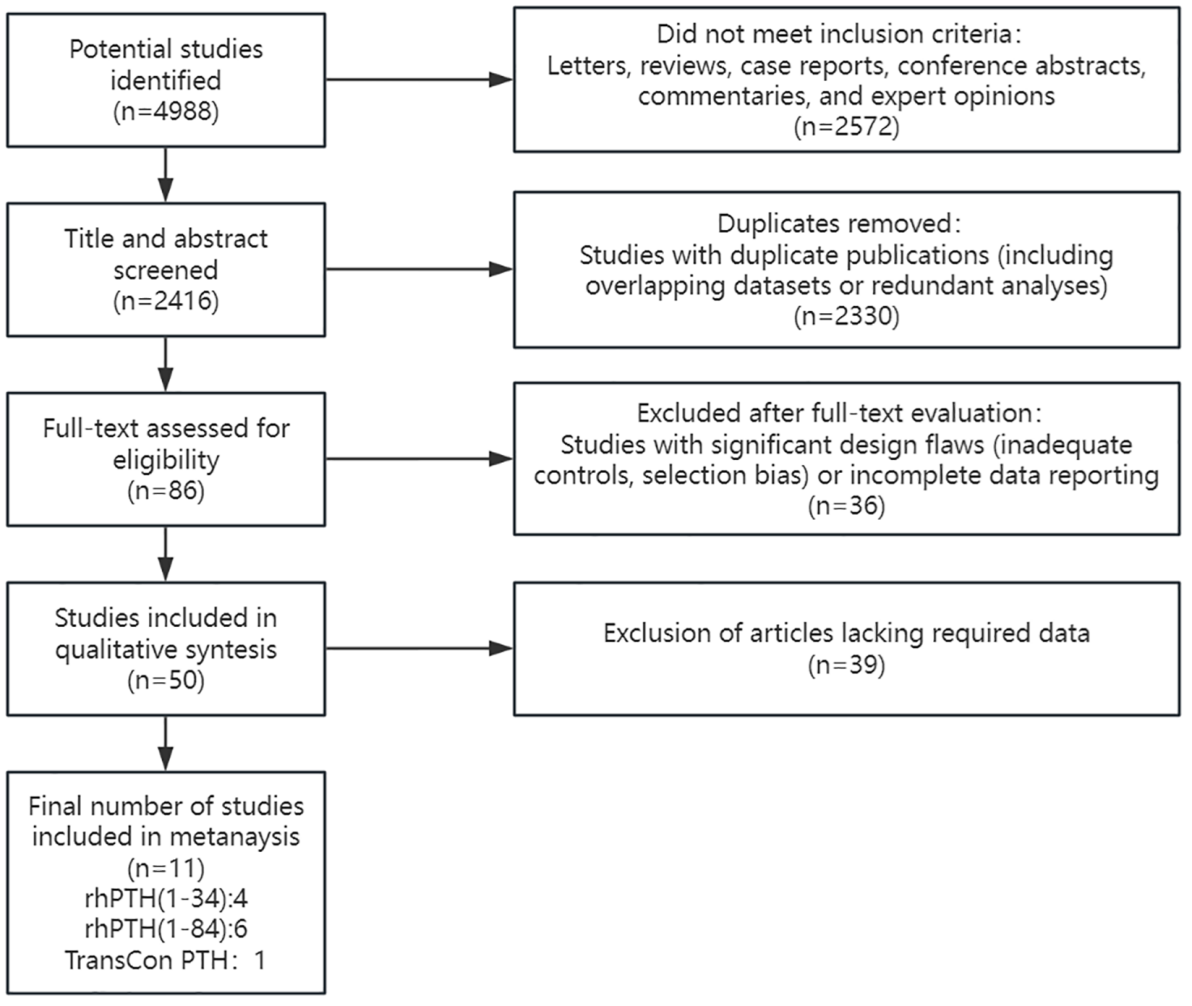
Flow chart for study selection.

A total of 4,998 relevant articles were initially retrieved. Of these, 3,113 were excluded after restricting the publication years and removing duplicates, and 1,835 were excluded after screening the abstracts and impact factors, leaving 50 articles for inclusion in this study. Subsequently, 39 articles were excluded after full-text assessment due to insufficient study design or lack of required data in this study. Ultimately, 11 studies were included in the meta-analysis, see **Figure 1**.

Therefore, this study analyzed 11 articles (4 on rhPTH (1-34), 6 on rhPTH (1-84), 1 on TransCon PTH (1-34)). These included 6 randomized controlled trials (RCTs), 4 open-label trials, and 1 historical controlled trial. A total of 672 patients were involved in this study, with study durations ranging from 6 to 96 months. Apart from the experimental groups in 6 RCTs, all studies included in this meta-analysis used calcium and active vitamin D as supplementary treatments. In RCTs comparing conventional treatment with PTH analogs treatment, the follow-up duration varied from 6 months to 60 months. The dosage of rhPTH (1-34) varied from 6 to 79.2μg daily, rhPTH (1-84) varied from 25 to 100μg daily, and TransCon PTH varied from 6 to 60μg daily. In studies examining the impact of PTH analogs on HRQoL in patients with HypoPTH, the follow-up duration ranged from 6 months to 96 months, and the PTH analogs dosage varied from 6 to 100 μg daily. The main characteristics of the studies are summarized in **Table 1**.

**Table 1:** Related studies for the period 1996-2025.

Year	First author	Study design	Center	Number of patients (rhPTH/control)	Etiology	Drug	Dosage and regimen	Study duration (months)	Main outcome (s)	Quality of study
2003	Winer (11)	RCT	NIH	27 (14/13)	PS:11; I:8; A:2; G:6	1‐34 vs conventional therapy	0.5μg/kg/dose (twice daily)	36	BMD Biochemistry Calcium and calcitriol supplementation	moderate
2010	Winer (12)	RCT	NIH	12 (7/5)	PS:0;I:5; A:4;G:1; NA:2	1‐34 vs conventional therapy	0.6 ± 0.5μg/kg (twice daily)	36	Biochemistry Adverse events	moderate
2013	Mannstadt (13)	RCT	Multicenter	134 (90/44)	PS:99; I:22; A:9; G:3; R:1	1–84 vs placebo	50–100μg/day	6	Biochemistry Adverse events Calcium and calcitriol supplementation	high
2014	Sikjaer (14)	RCT	Aarhus (Denmark)	62 (32/30)	PS:58; I:4	1–84 vs placebo	100μg/day	6	QOL Biochemistry Muscle function Postural stability	high
2017	Upreti (8)	open-label	New Dehli	8	PS:3; I:5	1‐34	Starting dose 20μg/dose (twice daily), subsequent individualized adjustment	18	BMD QoL Biochemistry Calcium and calcitriol supplementation	moderate
2018	Vokes (15)	RCT	Multicenter	122(83/39)	PS:87	1–84 vs placebo	Starting dose 50μg/day, subsequently increased to 100μg/day	6	QoL Biochemistry	high
2018	Palermo (10)	open-label	Multicenter	42	PS:42	1‐34	20μg/dose (twice daily)	24	QoL Biochemistry Adverse events Calcium and calcitriol supplementation	moderate
2019	Tabacco (16)	open-label	Columbia	20	PS:12; I:8	1‐84	Starting dose 100μg every other day, subsequently 25-75μg/day	96	QOL Biochemistry	moderate
2020	Chen (17)	historical controlled	Boston(USA)	122(69/53)	NA	1–84 vs conventional therapy	Starting dose 25 or 50μg/day, subsequently 25-100μg/day	60	Renal function	high
2023	Khan (18)	RCT	Hamilton(Canada)	84(63/21)	PS:70; I:7; A:2; G:3	TransCon PTH(1-34) vs placebo	Starting dose 18μg/day, subsequently 6-60μg/day	6.5	BMD QoL Biochemistry Adverse events Calcium and calcitriol supplementation	high
2024	Rubin (19)	open-label	Columbia	39	NA	1-84	25-100μg/day	35.45	BMD QoL Renal function Biochemistry Adverse events Calcium and calcitriol supplementation	moderate

A, autoimmune; BMD, bone mineral density; ECG, Electrocardiogram; G, genetic; I, idiopathic; NA, not available; PS, postsurgical; QoL, Quality of Life; RCT, Randomized Controlled Trial.

### Meta-analysis

#### The primary outcomes

##### Serum calcium

**Figure 2:**
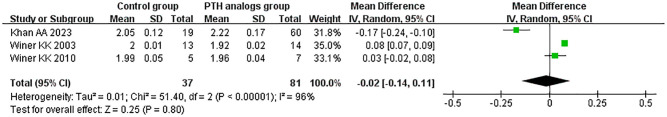
Comparison 1: conventional therapy versus PTH (1-34) analogs therapy on serum calcium levels.

Results from 3 RCTs of PTH (1-34) analogs with moderate heterogeneity observed no significant difference in serum calcium levels between the conventional therapy and the PTH analogs group (MD = -0.02 mmol/L; 95% CI, -0.14 to 0.11 mmol/L; p = 0.80), as illustrated in **Figure 2**.

##### Serum phosphate

**Figure 3:**
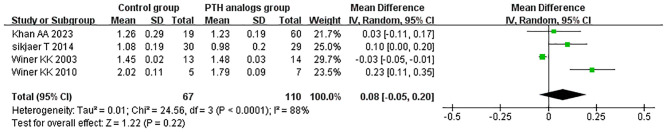
Comparison 2: conventional therapy versus PTH analogs therapy on serum phosphate levels.

There was no statistically significant difference in serum phosphorus levels (4 RCTs:2 on rhPTH (1-34), 1 on rhPTH (1-84) and 1 on TransCon PTH) between the PTH analogs therapy group and the control group (MD = 0.08 mmol/L; 95% CI, -0.05 to 0.20 mmol/L; p = 0.22), as illustrated in **Figure 3**.

#### The secondary outcomes

##### 24-hour urinary calcium excretion

**Figure 4:**
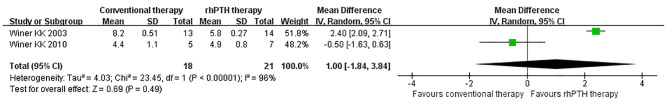
Comparison 3: conventional therapy versus PTH (1-34) analogs therapy on 24-hour urinary calcium excretion levels.

24-hour urinary calcium excretion was reported in 2 RCTs (11, 12) comparing conventional therapy with rhPTH (1-34). There was no statistically significant difference in 24-hour urinary calcium excretion levels (MD = 1.00 mmol; 95% CI, -1.84 to 3.84 mmol; p = 0.49), as shown in **Figure 4**.

#### Serum 25(OH)D and 1,25-dihydroxyvitamin D

**Figure 5:**
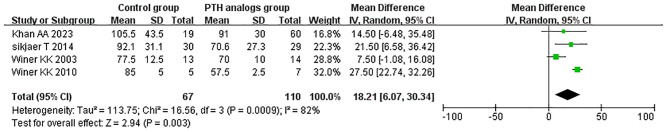
Comparison 4: conventional therapy versus PTH analogs therapy on serum 25(OH)D levels.

**Figure 6:**
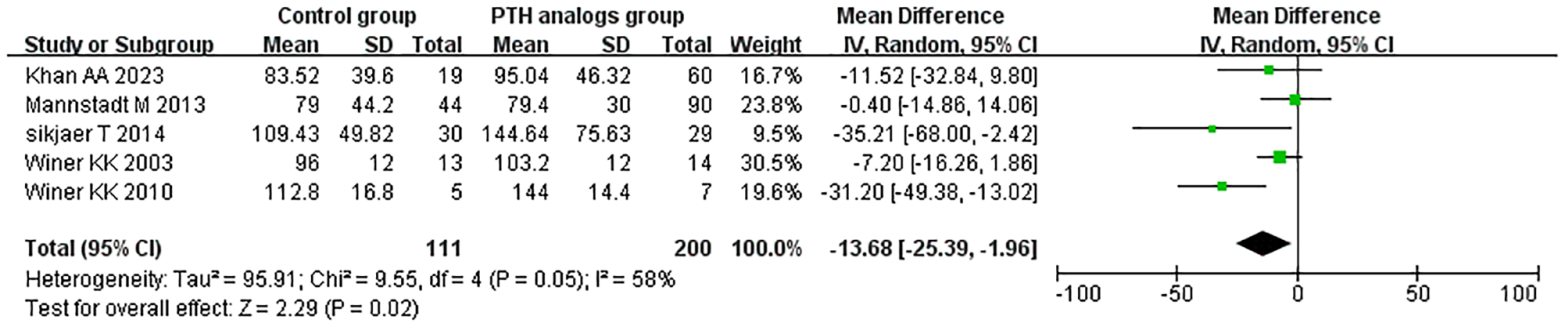
Comparison 5: conventional therapy versus PTH analogs therapy on serum 1,25-dihydroxyvitamin D levels.

The serum 25(OH)D levels in the PTH analogs treatment group were significantly lower than those in the control group (MD = 18.21 nmol/L; 95% CI, 6.07 to 30.34 nmol/L; p = 0.003; 4 RCTs (2 on rhPTH (1-34), 1 on rhPTH (1-84) and 1 on TransCon PTH), see **Figure 5**. The serum1,25(OH)_2_ vitamin D levels in the PTH analogs group were significantly higher than those in the control group (MD = -13.68 pmol/L; 95% CI, -25.39 to -1.96 pmol/L; p = 0.02; 5 RCTs (3 on rhPTH (1-34), 1 on rhPTH (1-84) and 1 on TransCon PTH), see **Figure 6**.

#### Calcium phosphate product

There was high heterogeneity between the two RCT studies, so the MDs were not pooled. The control group comprised 19 subjects, while the PTH analogs treatment group had 60 subjects in one study (18); 44 and 90 patients, respectively, in the other study (13). The calculated MD was -0.17 mmol (95% CI, -0.42 to 0.08 mmol) and 0.40 mmol (95% CI, 0.24 to 0.56 mmol).

#### Estimated glomerular filtration rate

**Figure 7:**
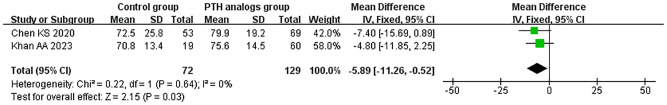
Comparison 6: conventional therapy versus PTH analogs therapy on eGFR.

eGFR in the PTH analogs group was significantly higher than that in the control group (MD = -5.89 ml/min/1.73m²; 95% CI, -11.26 to -0.52 ml/min/1.73m²; p = 0.03; 2RCTs), see **Figure 7**.

#### Adverse events

PTH analogs treatment-related adverse reactions are primarily mild to moderate in severity, with an overall favorable safety profile. The common adverse events include injection site reactions, hypercalcemia, hypercalciuria, hypocalcemia, neuromuscular symptoms (muscle cramps/pain, paresthesia, tetany), headache, nausea, and fatigue. Most adverse reactions can be alleviated through dose adjustment or discontinuation, without requiring hospitalization. Overall, no significant statistical difference in adverse reactions was observed between the two groups compared with the placebo group.

#### Health-related quality of life

**Figure 8:**
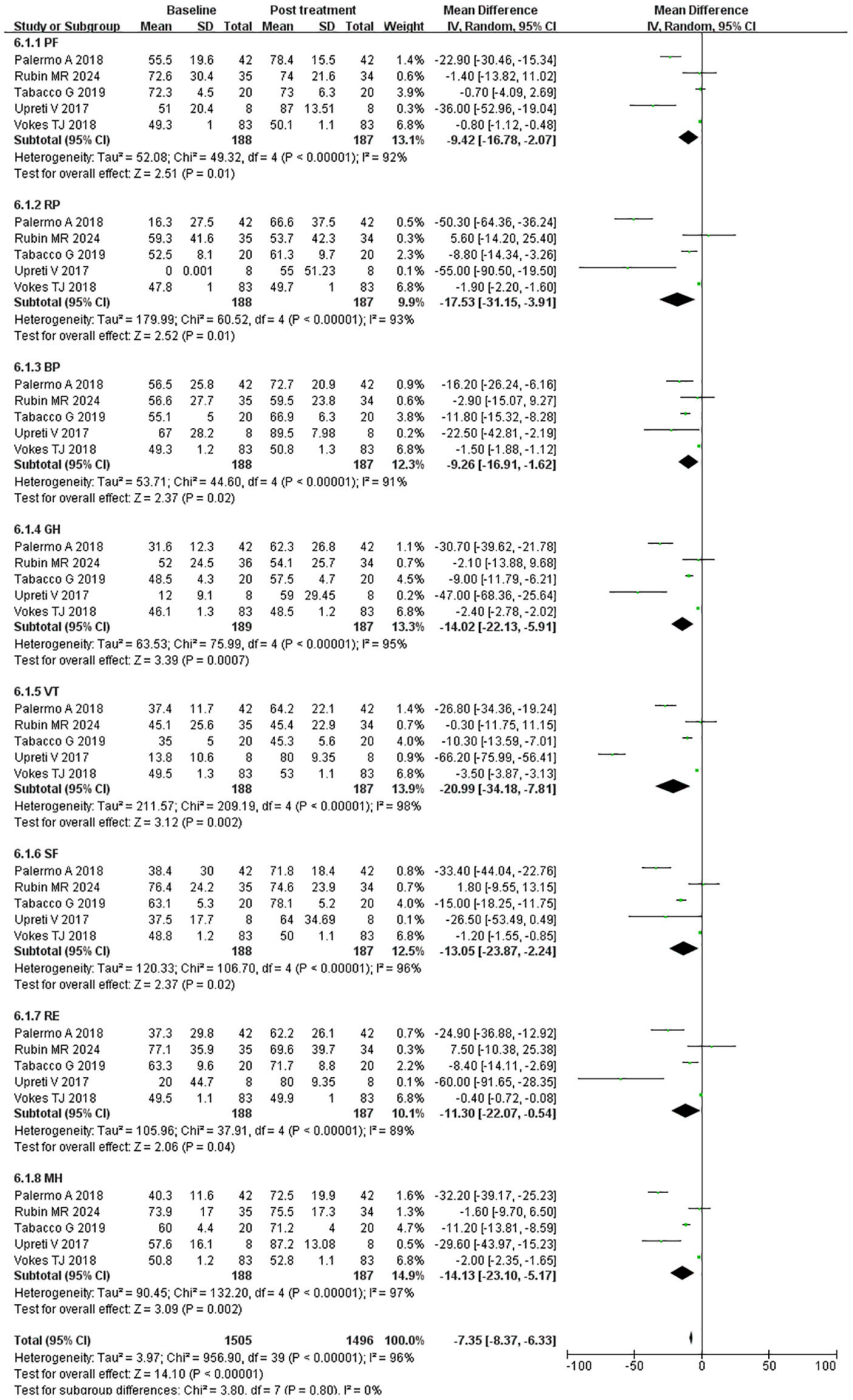
Patients’ scores on the SF-36 scale during PTH analogs therapy.

HRQoL before and after PTH analogs therapy was reported in 5 studies (188 patients), with 2 studies focusing on rhPTH (1-34) and 3 studies on rhPTH (1-84). PTH analogs treatment improved overall patients’ scores on the SF-36 scale (MD = -7.35; 95% CI, -8.37 to -6.33; p < 0.00001), and also in the subgroups in terms of the PF (MD = -9.42; 95% CI, -16.78 to -2.07; p = 0.01), RP (MD = -17.53, 95% CI, -31.15 to -3.91; p = 0.01), BP (MD = -9.26; 95% CI, -16.91 to -1.62; p = 0.02), GH (MD = -14.02, 95% CI, -22.13 to -5.91; p = 0.0007), VT (MD = -20.99, 95% CI, -34.18 to -7.81; p = 0.002), SF (MD = -13.05,95% CI, -23.87 to -2.24; p = 0.02), RE (MD = -11.30, 95% CI, -22.07 to -0.54; p = 0.04), and MH (MD = -14.13, 95% CI, -23.10 to -5.17; p = 0.002), respectively, see **Figure 8**.

## Discussion

Our meta-analysis indicated that PTH analogs therapy effectively regulates serum calcium by mimicking the physiological actions of endogenous PTH. However, heterogeneity was found in the included studies due to the study designs and different PTH administration regimens. Additionally, although occasional episodes of hypocalcemia or hypercalcemia occurred during PTH analogs treatment, their severity was significantly lower than that of conventional therapy. Patients with HypoPTH may develop hyperphosphatemia due to PTH deficiency, which consequently elevates the risk of complications such as kidney disease, cardiovascular disease, and neurological disorders. Significant benefit of serum phosphorus was not found yet in the PTH analogs treated HypoPTH as compared to the conventional treatment. This may be related to the multiple actions of PTH analogs. With short-term use, PTH analogs can promote bone resorption, during which the release of bone phosphorus may transiently increase blood phosphorus concentration. Additionally, PTH analogs can enhance the production of 1,25(OH)_2_D, as shown by our study, indirectly promoting intestinal absorption of phosphorus. When the balance between intestinal phosphorus absorption and renal phosphorus excretion is achieved, blood phosphorus levels may stabilize at a range similar to that observed with conventional therapy.

Conventional therapy for HypoPTH failed to restore PTH-mediated renal tubular calcium reabsorption, and there was a risk of developing hypercalciuria, thus leading to concerns about nephrolithiasis and nephrocalcinosis (3, 4). Therefore, it has created a clinical need for a medication that can manage urinary calcium excretion while treating HypoPTH. Some scholars have indicated that different dosing regimens of PTH analogs (once daily, twice daily, or continuous infusion via pump) significantly reduced 24-hour urinary calcium excretion in participants. The calcium phosphate product level is closely related to the risk of ectopic calcification in the body. Patients with HypoPTH, due to poor regulation of calcium and phosphorus metabolism, are exposed to the risk of ectopic calcification, while a study (13) has shown that PTH analogs can effectively mitigate such concerns. However, neither the conventional treatment group nor the PTH analogs treatment group in this meta-analysis reported the presence of heterotopic calcification. In addition, the data of MD on calcium phosphate product level were not pooled due to the limited sample size and the high heterogeneity between the studies (11–13, 18), which needs to be clarified by future studies. The insignificant difference in stabilizing calcium-phosphorus product concentrations was likely due to the nonsignificant differences in blood calcium and phosphorus levels treated by conventional therapy and PTH analogs in the study.

The eGFR is one of the core indicators for assessing renal function. The incidence of chronic kidney disease (CKD) in postoperative HypoPTH patients is three times that of the normal population, and six times higher in non-surgical patients, characterized by a progressive decline in eGFR (17). Two RCTs found that the eGFR was significantly higher in the PTH analogs group, indicating its renal-protective advantages. In this case, the risk of renal complications was reduced. In contrast, conventional treatment may accelerate the progression of kidney disease by increasing the renal filtration load, with some patients eventually developing end-stage renal disease requiring dialysis or kidney transplantation (9, 12, 20, 21).

In HypoPTH patients, common adverse events during the two treatments include fatigue, headache, paresthesia, muscle cramps and bone pain (11, 12, 14, 18), which are mostly associated with HypoPTH itself. Regarding metabolic aspects, hypocalcemia (12, 13) and hypercalcemia (18) are observed, but these could be effectively controlled by monitoring biochemical indices, adjusting supplement dosages, and optimizing PTH injection regimens. Moreover, long-term conventional treatment is prone to complications such as hyperphosphatemia, hypercalciuria, gastrointestinal reactions, basal ganglia calcification, kidney stones, and renal insufficiency (11). However, due to differences in the description of adverse events across studies, we do not perform a data comparative analysis. There remains controversy over whether rhPTH (1-34) increases the risk of osteosarcoma. Among more than 400,000 treated patients, only two cases of osteosarcoma have been reported, a rate much lower than the epidemiological expectation. Large-sample and long-term follow-up studies are needed to clarify the long-term adverse effects of the drug and the association between rhPTH (1-34) and osteosarcoma.

HypoPTH not only disrupts biochemical parameters but also exerts multidimensional negative impacts on patients’ physiological functions, psychological status, and social well-being. Patients had poor Short-Form 36-Item Health Survey (SF-36) scores under standard treatment, whereas PTH analogs comprehensively improved HRQoL, which may be attributed to the relief of hypocalcemia and its consequent pain and negative emotions (22). Subgroup analysis revealed that the rhPTH (1-34) subgroup showed more significant improvements in the PF, RP, BP, and GH dimensions. The differences in therapeutic effects between formulations may stem from the heterogeneity of study designs or the potential influence of molecular bioactivity. However, no study was found to compare the effects of conventional therapy versus PTH analogs on the quality of patients’ lives, which also needs to be clarified.

Currently, PTH analogs treatment mainly relies on subcutaneous injection, which are more cumbersome compared to the oral administration of calcium and active vitamin D. Additionally, the cost of PTH analogs is significantly higher than that of calcium and active vitamin D supplements, imposing a heavy economic burden on HypoPTH patients. Therefore, in terms of accessibility, convenience, and cost, conventional treatment is superior to PTH analogs treatment. However, when patients experience poor disease control with conventional treatment or require high doses of supplements, PTH analogs treatment may be a viable option.

Apart from the three PTH analogs in this study, the novel agent eneboparatide has entered clinical research. As a long-acting PTH1 receptor agonist consisting of a 36-amino-acid peptide, it selectively binds the R0 conformation of PTH1 receptor, producing sustained effects despite a short half-life, and is given by once-daily subcutaneous injection. Clinical trials show it stabilizes serum calcium, reduces urinary calcium excretion, and allows most hypoparathyroidism patients to stop calcium and active vitamin D supplements (23). It improves renal function, maintains normal bone metabolism and has a good safety profile. Its Phase II trials are completed and the multinational Phase III CALYPSO trial is ongoing (24). Not yet approved for clinical use, it requires further data to confirm its long-term efficacy, safety and real-world application.

The meta-analysis has limitations in several aspects. The sample sizes of the included studies are relatively small. Additionally, differences in patients’ baseline characteristics (patient age, etiology, and disease duration) and significant heterogeneity in outcome measurements across studies reduce the general applicability of the conclusions. Among these, variations in detailed dosing regimens and administration methods are the key factors contributing to the heterogeneity of outcomes. However, due to the limited number of eligible studies and incomplete reporting of these variables, we were unable to obtain sufficient data to perform subgroup analyses based on these factors.

Moreover, most studies have a short observation duration, and there is a lack of favorable evidence for comparing the long-term efficacy, safety, and drug resistance of hormone replacement therapy. Therefore, it is necessary to conduct future studies with large-scale samples and long-term follow-up to clarify the long-term advantages of PTH analogs. Future research could focus on dynamic monitoring of biochemical indicators throughout the entire period, rather than relying on data from specific time points. Renal function may be evaluated not only by eGFR, but also the serum creatinine, blood urea nitrogen, uric acid, and urinary microalbumin.

## Conclusion

In addition to the comparable control of serum calcium and serum phosphorus levels to conventional calcium and calcitriol supplements therapy, limited data indicate that PTH analogs treatment may be better in regulating the serum vitamin D levels and maintaining the eGFR for patients with HypoPTH. Therefore, PTH analogs has the potential to reduce the risk of complications caused by long-term and high-dose calcium and calcitriol supplements and protect renal function, which needs to be clarified by a well-designed clinical trial. PTH analogs therapy also improves patients’ HRQoL. The subgroup results of PTH (1-34) analogs and PTH (1-84) analogs, and the long term adverse events of PTH analogs also need further study.

## Data Availability

The original contributions presented in the study are included in the article/supplementary material. Further inquiries can be directed to the corresponding author.
